# Emotional Intelligence, Perceived Academic Self-Efficacy, and Perfectionistic Automatic Thoughts as Predictors of Aesthetic-Musical Awareness in Late Adolescence

**DOI:** 10.3389/fpsyg.2021.733025

**Published:** 2021-08-12

**Authors:** Rosa Pilar Esteve-Faubel, María Pilar Aparicio-Flores, Victoria Cavia-Naya, José María Esteve-Faubel

**Affiliations:** ^1^Department of General Didactics and Specific Didactics, University of Alicante, Alicante, Spain; ^2^Department of Didactics of Music, Plastic and Corporal Expression, University of Valladolid, Valladolid, Spain

**Keywords:** aesthetic-musical awareness, aesthetic desire, emotional intelligence, academic self-efficacy, perfectionistic automatic thoughts

## Abstract

Aesthetic-musical awareness demarcates a person's own perception of their ability to connect with music and the emotions it evokes. This may imply a benefit for the affective state of the individual. Therefore, the aim of this study was to observe whether there are statistically significant differences in emotional intelligence, perceived academic self-efficacy, and perfectionistic automatic thoughts when there are high and low scores in aesthetic-musical awareness in late adolescence. Likewise, we also aimed to determine whether emotional intelligence, perceived academic self-efficacy, and perfectionistic automatic thoughts are predictors of high aesthetic-musical awareness. To this end, a sample of 798 Spanish students between 17 and 23 years of age (*M*_age_ = 18.5 years) was used. Statistically significant differences were found for the dimensions of each variable when there were high and low scores in aesthetic-musical awareness (scores between *d* = −0.31 and −0.40), with higher mean scores for emotional intelligence, perceived academic self-efficacy, and perfectionistic automatic thoughts being present in the group with high aesthetic-musical awareness. Likewise, it was observed that the probability of presenting high scores in aesthetic-musical awareness was higher when there was an increase in emotional intelligence, perceived academic self-efficacy, and perceived automatic thoughts. In conclusion, the results found demonstrate that both emotional intelligence, perceived academic self-efficacy, and the presence of perfectionistic automatic thoughts influence on whether an individual has greater aesthetic-musical awareness. Taking into account previous studies that show how music influences the well-being of the person, these findings show a favorable link for the design of programs that benefit the emotional state of adolescents.

## Introduction

Awareness, attention, and understanding of emotions are essential to accurately identify a person's emotional state (Chong-Leung and Cheung, 2020). This directly influences an individual's quality of life (Boden et al., 2015) and mental health (Lieberman et al., 2011), especially in the case of adolescence (Ciarrochi et al., 2011), which is characterized as a stage in which very intense emotions are experienced, but in which the skills to regulate them are not yet fully developed (Dingle et al., 2016).

Research that focuses on the emotions evoked by music is increasing due to the great adaptive power that music has for humans not only on an emotional level but also in terms of attachment and social and cultural belonging (Hunter and Schellenberg, 2010; Fritz et al., 2013; Koelsch, 2018, 2020; Ehrlich et al., 2019; Varner, 2019). Music is a medium that amplifies and alters emotions, especially for adolescents (Chong-Leung and Cheung, 2020). It influences emotion regulation (Stewart et al., 2019), while generating emotional well-being and increased awareness (Dingle et al., 2016).

According to Koelsch (2018), music can arouse different emotional, cognitive, and physiological components. These emotional components are interrelated with high Aesthetic-Musical Awareness (AMA), which is a dimension of Aesthetic Desire that is also known as one's self-awareness of the ability to connect with music and the emotions it evokes (Aparicio-Flores et al., 2020a). In other words, the pleasurable affect generated by music is linked to deeper psychophysiological and cognitive aspects, ranging from emotion regulation to emotion arousal (Saarikallio et al., 2018). However, in this process, there is a conscious step involved which is the AMA.

In terms of its general conceptualization, Aesthetic Desire is understood as the emotional sensitivity that generates pleasant stimuli when observing or creating a work of art (Lundy et al., 2010; Aparicio-Flores et al., 2020a). This is a variable that has been recently studied and intervenes with the affective processes generated by plastic or architectural works of art, the beauty of one's surroundings, and musical works (Koelsch, 2018; Aparicio-Flores et al., 2020a).

Nowadays, the study of Aesthetic Desire is encouraged due to its relevance in aspects such as the frequency with which it is generated in an individual's day-to-day life and the psychological well-being it can promote for them (Saarikallio et al., 2018). The need to further understand this construct is also implied considering that a work of art, in this case pertaining to music, can be evaluated as something positive even though it triggers a negative feeling (e.g., melancholy) (Brattico et al., 2016; Koelsch, 2018). Taking this into account, it is relevant to inquire into people's AMA in order to observe whether this allows for the design of effective strategies for the regulation of an individual's emotions.

Being aware of one's feelings is greatly significant given the benefits that it can bring both at the interpersonal and intrapersonal levels (Karimi, 2021). Therefore, having high Emotional Intelligence (EI) is important because this is the ability to perceive, understand, and regulate one's emotions (Salovey and Mayer, 1990). As such, observing the link between EI and AMA can be of significance for people's daily lives, as well as for educational and therapeutic purposes.

Emotion is an affective experience that prepares humans to adapt to a particular environment, with its basic function being survival (Lorenzo-de Reizábal, 2019). Several studies claim that a change in emotional state can be clearly noted when listening to music, without this being conditional to whether the listener has had previous musical experience or even whether the individual has been governed by a particular personality trait (Mohn et al., 2011; Saarikallio et al., 2018; Lorenzo-de Reizábal, 2019). In fact, some functional neuroimaging studies claim that music acts on various brain structures that have direct involvement with emotions, such as the hippocampus, hypothalamus amygdala, and auditory cortex, among others (Koelsch, 2014, 2020; Koelsch and Skouras, 2014).

However, in the development of the teaching-learning process regarding emotional competence, and consequently EI, learning to be aware of one's own emotions is established as a fundamental step (Mayer et al., 2000; Lorenzo-de Reizábal, 2019). In this regard, authors such as Bisquerra (2017) note that listening to music is a way to allow this process to happen since at the same time that one discovers that an emotion has been evoked, they regulate it, which implies that it can be of great use for emotional education.

It seems as though EI is related to music listening (Londsdale, 2018). Many people listen to music to control their emotional state (Londsdale and North, 2011). To this end, these individuals must have a greater level of recognition and management of their own emotions. In other words, they need to have developed EI, with the use of music depending, in part, on the level of EI acquired (Londsdale, 2018).

However, for most people, music is a recreational and motivating activity, in which the emotions experienced while listening are not consciously analyzed (Lorenzo-de Reizábal, 2019). In fact, music can generate different emotions depending on the person listening to it (Kawakami et al., 2013), and even if the same emotion is felt, it can be experienced with different intensities (Schubert, 2013). For this reason, music does not only influence the reflection of the emotions felt but also can serve as a motivational strategy, a source of enjoyment (Lorenzo-de Reizábal, 2019), a tool to regulate emotions whatever the intensity (Stewart et al., 2019), and even as a verbal tool for the communication of such emotions (Kragness and Trainor, 2019).

Considering the role that music can have in triggering EI, and that EI is decisive for people's well-being socially (Nikooyeh et al., 2017), emotionally (Bucich and MacCann, 2019), and cognitively (Nirmala-Wijekoon et al., 2017), as well as it also being relevant at adolescence due to the characteristics of the stage and the search for one's own identity (Bucura, 2019), it is important to analyze the connection between EI and AMA in late adolescence.

On the other hand, this can be important for adolescence, being that it is a stage in which young people are immersed in their studies and are reflecting on their interests and the cognitive abilities that will improve their future career. In this sense, it is worth highlighting Academic Self-Efficacy (ASE), which is understood as one's belief about their own ability to achieve a goal, in this particular case, academic goals (García-Fernández et al., 2016b; Yokoyama, 2019) This may contribute to higher academic performance (García-Fernández et al., 2016a; Honicke and Broadbent, 2016; Yokoyama, 2019) and the adoption of metacognitive strategies (Asghar-Hayat and Shateri, 2019), as well as dispositional empathy (Aparicio-Flores et al., 2020b), motivation (Ross et al., 2016), and academic resilience (Cassidy, 2015). It is therefore valuable to observe the relationship between ASE and AMA.

The essential external factors that affect individuals are aspects such as education, employment, motivation, and the need for a sense of achievement, among others (Hasanuddin and Sjahruddin, 2017). As such, it is important to obtain a high level of ASE that influences academic performance (Honicke and Broadbent, 2016; Yokoyama, 2019), as well as emotional and social aspects (Bucura, 2019; Aparicio-Flores et al., 2020c). In fact, it should be considered that a person's performance is not only observed in terms of their work capacity but also regarding their self-control, and even social relationships (Hasanuddin and Sjahruddin, 2017).

It has been shown that it is possible to regulate and control people's behavior in learning contexts (Agina et al., 2011). However, this depends on the pedagogical approach employed by educational professionals (Fernández-Rio et al., 2017). It has also been observed that the higher the self-regulated learning in students, the higher their ASE (Fernández-Rio et al., 2017). As such, there is a certain significance in nurturing students with strategies that promote the regulation of their own learning and behavior.

Adolescents tend to lead a rich and active musical life (Campbell et al., 2007). However, this does not imply that they possess a high level of efficacy in their music lessons. This is where ASE, among other components, influences the final outcome (Bucura, 2019).

Background music as a learning style, although it does not influence ASE, has a positive impact on the students' sense of achievement, bringing happiness, motivation, and greater enjoyment for a task, which has an impact on their academic success (Can and Güven, 2020) and results in less mental effort and time spent on the task (Lesiuk, 2005; Lim and Bang, 2018). Similarly, musical training influences emotional perception and mental imagery capacity, which contributes to academic learning (Commodari and Sole, 2019). Moreover, musical activity facilitates social bonding among students, promotes tolerance to diversity, and motivation for learning (Cores-Bilbao et al., 2019).

Considering the benefits of musical listening at the level of academic performance, and that musical pleasure comprises feelings of relaxation, joy, and power (Saarikallio et al., 2018), and without observing studies that analyze the direct link between AMA and ASE, it would be relevant to analyze the role of AMA on ASE in late adolescence.

Likewise, it should be considered that the demands of our current society are governed by perfect standards. In this regard, the adolescent stage may be conditioned to follow a pattern based on excellence at any level (e.g., physical, academic, social). As such, special attention should be paid to Perfectionistic Automatic Thoughts (PATs), which are understood as automatic reflections that are based on the desire to be perfect (Flett et al., 1998, 2012; Esteve-Faubel et al., 2020). These thoughts have a negative influence at the academic (Desnoyers and Arpin-Cribbie, 2015; Flett et al., 2019), physical (Flett et al., 1998, 2012; Kirtley et al., 2015), social (Aparicio-Flores et al., 2020c), and emotional level (Flett et al., 1998; Macedo et al., 2017; Aydin and Yerin-Güneri, 2020; Esteve-Faubel et al., 2020; Tyler et al., 2021). For this reason, it would be necessary to reflect on the connection that exists between AMA and PATs in order to determine whether AMA can positively influence strategies that enhance these perfectionistic reflections.

Perfectionism is known as a personality trait that negatively affects people who suffer from it given that it is determined by the establishment of very high and immoderate standards for the achievement of a proposed goal (Smith et al., 2021). Deriving from the more cognitive part, PATs are about specific ruminations of perfectionism (Flett et al., 2012).

A person with perfectionistic traits tends to demand an excessive quality of performance from themself in any task, therefore generating high levels of anxiety (Sarikaya and Kurtaslan, 2018). In this regard, some musicians have not been able to give any scheduled concerts throughout their professional life due to their high levels of anxiety (Kenny, 2011).

However, while music performances can generate high levels of stress and anxiety due to PATs (Flett et al., 2016; Aydin and Yerin-Güneri, 2020; Tyler et al., 2021), several studies claim that people with high levels of stress or emotionally negative experiences tend to listen more to music as a way of controlling their mood (Getz et al., 2012, 2014) because music listening has the power to decrease tension, anxiety, and stress (Lim and Bang, 2018). In other words, performing, and doing so on a crowded stage, is not the same as listening to music with the purpose of achieving a state of relaxation. Similarly, neither music performance nor listening, which have a positive or negative impact on the anxiety caused by perfectionism (Flett et al., 1998; Sarikaya and Kurtaslan, 2018; Aydin and Yerin-Güneri, 2020; Tyler et al., 2021), are the same as AMA.

There is little research based on perfectionism and music, and there is even less concrete research on the link between music and PATs. However, despite observing some research that supports this positive or negative link, depending on the case to be examined, there is a lack of research showing related findings between PATs and AMA.

Given the characteristics of AMA, which generate a specific internal and emotional knowledge of a person, it could have a beneficial influence on the design of strategies for the prevention of PATs and the regulation of other emotions of negative affect. Therefore, it is important to observe how AMA influences the PATs of the individual who presents them.

### In the Current Study

The present study aims to: (a) analyze whether there are statistically significant differences in EI, ASE, and PATs in relation to high and low AMA scores in late adolescence; and (b) determine whether EI, ASE, and PATs are predictors of high AMA.

Taking into account the previous literature, it is expected that:

- *Hypothesis 1*: there are statistically significant differences in EI based on high and low AMA scores in late adolescence, and that EI is a predictor of high AMA (Mohn et al., 2011; Koelsch, 2014, 2020; Koelsch and Skouras, 2014; Saarikallio et al., 2018; Lorenzo-de Reizábal, 2019).
- *Hypothesis 2*: there are statistically significant differences in ASE based on high and low AMA scores in late adolescence, and that ASE is a predictor of high AMA (Lesiuk, 2005; Lim and Bang, 2018; Can and Güven, 2020).
- *Hypothesis 3*: there are statistically significant differences in PATs based on high and low AMA scores in late adolescence, and that PATs are predictors of high AMA (Getz et al., 2012, 2014; Lim and Bang, 2018).

## Method

### Participants

The participant sample was recruited under the accessibility criteria in the Faculty of Education at the University of Alicante.

A total of 824 students were selected. These were students who were in either their first or second academic year, and were studying degrees in either Early Childhood Education or Primary Education. The inclusion criteria were that the participants were future teachers so that the results are known to all of them and can apply interesting aspects in their future classrooms.

Twenty-two (2.67%) participants were excluded due to errors or omissions in the completion of the questionnaires, these being the only reasons for exclusion. Therefore, the final sample of 798 participants was achieved, with ages ranging between 17 and 23 years (*M*_age_ = 18.5; *SD* = 3.2), and with 70.3% being women.

Regarding the ethnicity of the participants, it should be noted that the vast majority of students were Spanish (92.23%). However, 3.88% were European, 2.01% Arab, 1.00% South American and 0.88% Asian. However, all of them had been living in Spain for more than 7 years.

### Instruments

**Trait Meta-Mood Scale 24** (TMMS-24; Fernández-Berrocal et al., 2004): The TMMS-24 is a Likert-type scale with 5 response points (1 = *Not agree at all*; 5 = *Strongly agree*) that assesses EI from 24 items and three dimensions: I. Attention to feelings (e.g.: I pay close attention to feelings); II. Mood repair (e.g.: I can come to understand my feelings); and III. Clarity of feelings (e.g.: When I'm sad I think of all the pleasures in life).

Reliability levels of the scale were acceptable for all dimensions (α = 0.89, 0.90, and 0.85, respectively).

**Academic Situations Specific Perceived Self-Efficacy Scale** (SPSASS; García-Fernández et al., 2010). The Spanish version of the SPSASS, adapted from the original scale by Palenzuela (1983), is a Likert-type scale with four response options (1 = *Never*; 4 = *Always*), consisting of 10 items and a unidimensional structure. This scale measures self-efficacy expectations in educational situations for both high school and university students (e.g.: I think I can get through a semester quite easily, and even with very good grades).

The internal consistency of the instrument for this study was α = 0.88.

**Perfectionism Cognitions Inventory** (PCI; Esteve-Faubel et al., 2020): The Spanish version of the PCI is an adaptation of the work done by Flett et al. (1998), which assesses PATs. The PCI is a Likert-type scale with 5 response options (1 = *Not at all*; 5 = *All the time*) and is composed of 17 items that make up three dimensions and a second-order factor. These dimensions measure: I. Perfectionistic Concerns (e.g.: I certainly have high standards); II. Perfectionistic Demands (e.g.: I've got to keep working on my goals); and III. Perfectionistic Strivings (e.g.: I expect to be perfect).

Reliability levels were acceptable for all 3 dimensions in the present study (α = 0.83, 0.71, 0.86, respectively).

**Desire For Aesthetic Scale** (DFAS; Aparicio-Flores et al., 2020a): The Spanish version of the DFAS is an adaptation of the original version designed by Lundy et al. (2010), which assesses Aesthetic Desire. The DFAS is a Likert-type scale with seven response options (1= *Strongly Disagree*; 7 = *Strongly Agree*) and 11 items configured in three dimensions: I. Satisfaction with Aesthetic Beauty (SAB) (e.g.: In the past, when I've mod into a new apartment, office or dorm, one of the first things I do is decorate the walls with nice artwork); II. Aesthetic-Musical Awareness (AMA) (e.g.: Hearing gorgeous songs is a major motivator for me in daily life); III. Emotional State of Aesthetic Beauty (ESAB) (e.g.: I would not to be happy in an unattractive house).

Specifically, in this study, the AMA dimension was administered, which obtained adequate reliability indices (α = 0.72).

### Procedure

Firstly, a meeting was held with the teachers of the subjects in which the study questionnaires were to be administered, in order to inform them of the objectives and request their collaboration. Later, in another session, the participants were informed of the purpose of the study, they were asked for their informed consent, and they were told about their status as volunteers and about their anonymity.

Subsequently, the different tests were administered in the classroom within an estimated time of 45 min, during which a researcher was present.

The research was carried out under the recommendations of the ethical principles of the Declaration of Helsinki of 1964.

### Data Analysis

Firstly, a Student's *T*-test was applied to study the differences in EI, ASE, and PATs according to the high and low AMA scores. To evaluate the magnitude of the differences found, Cohen's (Cohen, 1988) d index was included.

Secondly, to determine the predictive probability of EI, ASE, and PATs on high AMA, the statistical technique of logistic regression was applied, following the forward stepwise procedure based on the Wald statistic. The probability was estimated through the odds ratio (OR) in accordance with the following interpretation: if OR = > 1, for example, OR = 2, for each occurrence of the situation in the presence of the independent variable, there will be two times the variable present. If, on the other hand, OR = <1, for example, OR = 0.5, the probability of that situation occurring in the absence of the independent variable will be 0.5 times lower than in its presence.

For the analysis of the quality and fit of the proposed models, the following indicators were considered: Nagelkerke's R^2^, which indicates the percentage of variance explained by the model (Nagelkerke, 1991); and the diagnostic efficiency, that is, the percentage of cases correctly classified by the model.

It should be noted that AMA was dichotomized according to low scores (percentile = > 25) and high scores (percentile = <75).

## Results

### Differences in Emotional Intelligence, Academic Self-Efficacy, and Perfectionistic Automatic Thoughts in Late Adolescence With High and Low Scores in Aesthetic-Musical Awareness

Table 1 shows the statistically significant differences obtained in EI, ASE, and PATs for late adolescence in accordance with high and low AMA scores. The observed differences were statistically significant, and were also of small magnitude, for all EI, PATs, and ASE factors. Thus, the results show that all three EI factors, attention to feelings (*M* = 28.20; *SD* = 6.55), mood repair (*M* = 27.04; *SD* = 6.72) and clarity of feelings (*M* = 26.49; *SD* = 6.39), score higher in the high AMA scores compared with the low AMA group.

**Table 1:** Differences in emotional intelligence, academic self-efficacy, and perfectionistic automatic thoughts in late adolescence according to high and low scores in aesthetic-musical awareness.

	**Levene's test**		**Low scores**		**High scores**		**Statistical significance**			
**Dimensions**	***F***	***p***	***M***	***SD***	***M***	***SD***	***t***	***g.l*.**	***p***	***d***
A_TMMS	0.88	0.34	26.08	6.14	28.20	6.55	−3.37	424	0.001	−0.33
M_TMMS	5.53	0.01	24.85	6.10	27.04	6.72	−3.40	401.50	0.001	−0.34
C_TMMS	0.21	0.64	24.50	6.46	26.49	6.39	−3.14	424	0.002	−0.31
ASE_SPSASS	4.84	0.02	29.97	5.59	32.25	6.63	−3.82	413.06	<0.001	−0.37
PC_PCI	4.43	0.03	14.01	4.83	15.71	5.46	−3.32	405.92	0.002	−0.33
PD_PCI	7.17	0.01	13.52	3.20	14.68	2.60	−3.96	331.49	<0.001	−0.40
PS_PCI	0.00	1.00	22.08	5.51	23.83	5.31	−3.28	424	0.001	−0.32

*A_TMMS, attention to feelings; M_TMMS, mood repair; R_TMMS, clarity of feelings; ASE_ SPSASS, academic self-efficacy; PC_PCI, perfectionistic concerns; PD_PCI, perfectionistic demands; PE_PCI, perfectionistic strivings*.

Likewise, higher ASE scores are obtained for the group with high scores as opposed to the group with low AMA scores (*M* = 32.25; *SD* = 6.63).

On the other hand, the high AMA profile vs. the low AMA profile obtained higher scores in perfectionistic concerns (*M* = 15.71; *SD* = 5.46), perfectionistic demands (*M* = 14.68; *SD* = 2.60), and perfectionistic strivings (*M* = 23.83; *SD* = 5.31).

### Predictive Capacity of the Variables Emotional Intelligence, Academic Self-Efficacy, and Perfectionistic Automatic Thoughts on Aesthetic-Musical Awareness

As can be seen in Table 2, two logistic regression models have been created from which correct estimates can be made regarding the probability of showing high AMA considering EI, ASE, and PATs scores.

**Table 2:** Binary logistic regression for the probability of presenting high scores in aesthetic musical awareness based on emotional intelligence, academic self-efficacy, and perfectionistic automatic thoughts.

**Variable**		*****χ^2^*****	***R^**2**^***	***B***	***E.T*.**	**Wald**	***p***	***OR***	**I.C. 95%**
A_TMMS	Classified correc.: 58.7%	11.24	0.04	0.05	0.01	10.90	0.001	1.05	1.02–1.09
	Constant		−1.07		0.43	6.09	0.014	0.34	
M_TMMS	Classified correc.: 61.2%	11.82	0.05	0.06	0.01	11.01	0.001	1.06	1.03–1.09
	Constant		−0.92		0.44	5.83	0.025	0.40	
C_TMMS	Classified correc.: 61%	9.87	0.03	0.05	0.01	9.53	0.002	1.05	1.02–1.08
	Constant		−0.90		0.41	4.88	0.027	0.40	
ASE_ SPSASS	Classified correc.: 58.7%	13.70	0.04	0.05	0.01	13.00	<0.001	1.06	1.03–1.09
	Constant		−1.51		0.51	8.52	0.004	0.22	
PC_PCI	Classified correc.: 60.4%	14.37	0.04	0.11	0.04	12.3	<0.001	1.07	1.03–1.11
	Constant		−1.24		0.30	12.4	0.001	0.28	
PD_PCI	Classified correc.: 60.6%	16.48	0.05	0.14	0.03	15.6	<0.001	1.15	1.07–1.23
	Constant		−1.63		0.50	10.5	0.001	0.19	
PS_PCI	Classified correc.: 59.6%	10.67	0.03	0.06	0.01	10.30	0.001	1.06	1.02–1.10
	Constant		−1.04		0.43	5.69	0.017	0.35	

*Classified correc., classified correctly; A_TMMS, attention to feelings; M_TMMS, mood repair; C_TMMS, clarity of feelings; ASE_ SPSASS, academic self-efficacy; PC_PCI, perfectionistic concerns; PD_PCI, perfectionistic demands; PE_PCI, perfectionistic strivings*.

The findings allow us to make a correct estimate for attention to feelings in 58.7% of cases (χ^2^ = 11.24; *p* = 0.001). These data allow us to affirm that this dimension of EI is a predictor of high AMA. The components of the model revealed by the odds ratio (OR) indicate that the probability of presenting high levels of AMA is greater, specifically 1.05 for each point increase in the attention to feelings factor of EI. Likewise, with respect to mood repair, the data allow us to make a correct estimate for 61.2% of cases (χ^2^ =11.82; *p* = 0.001), indicating that this EI factor is also a predictor of AMA. The components of the model showing the odds ratio (OR) indicate that the probability of having high levels of AMA is 1.06 greater for each point that mood repair increases. Regarding clarity of feelings, the results found allow us to make a correct estimate in 61% of cases (χ^2^ = 9.87; *p* = 0.002), suggesting that this dimension of the TMMS-24 is a predictor of a high level of AMA. The components of the model showing the odds ratio (OR) indicate that the probability of presenting a high AMA is 1.05 higher for each point increase in clarity of feelings.

On the other hand, with respect to the variable of ASE, the data allow for a correct estimation in 58.7% of cases (χ^2^ =13.70; *p* = < 0.001), indicating that ASE is a predictor of high levels of AMA. In this case, the components of the model that show the odds ratio (OR) indicate that the probability of showing a high level of AMA is 1.06 higher for each point that ASE increases.

Finally, regarding PATs, the data concerning perfectionistic concerns offer a correct estimate in 60.4% of cases (χ^2^ =14.37; *p* = < 0.001), suggesting that this dimension of PATs is predictive of high AMA. The components of the model showing the odds ratio (OR) highlight that the probability of presenting high AMA is 1.07 higher for each point that perfectionistic concerns increase. Similarly, with respect to perfectionistic demands, the results obtained allow for a correct estimation in 60.6% of the cases (χ^2^ =16.48; *p* = < 0.001), indicating that this factor of the PATs is a predictor of high levels of AMA. The components of the model showing the odds ratio (OR) indicate that the probability of presenting high levels of AMA is 1.15 higher for each point increase in perfectionistic demands. Likewise, in relation to perfectionistic strivings, a correct estimate can be made for 59.6% of cases (χ^2^ =10.67; *p* = 0.001), suggesting that this factor of PATs is a predictor of high AMA. The components of the model showing the odds ratio (OR) indicate that the probability of having a high AMA is 1.06 higher for each point that perfectionistic strivings increase.

## Discussion

The findings of this study indicate statistically significant differences for all EI, ASE, and PATs factors when considering high and low AMA scores, showing higher scores in the group with high AMA in all cases. Similarly, it is observed that EI, ASE, and PATs are predictors of high AMA.

Regarding EI, the results show that the three EI factors, attention to feelings, mood repair, and clarity of feelings score higher in the group with a high AMA level compared to the profile with a low level of AMA. Likewise, all three dimensions of EI are predictors of high AMA. Therefore, all the hypotheses of the study are fulfilled.

Possessing high EI demonstrates a better perception and understanding of the emotional state the person is going through at a given moment (Salovey and Mayer, 1990; Mayer et al., 2000; Lorenzo-de Reizábal, 2019). Therefore, this ability to perceive emotions can also be determined by contexts in which music is the key to the altering of emotions. In other words, this means having the ability to perceive, understand, and regulate one's own emotions (these being the three factors of EI) whether from music or from any other context.

It is worth remembering that music has the ability to alter and amplify emotions (Chong-Leung and Cheung, 2020), while at the same time regulating them (Bisquerra, 2017; Londsdale, 2018; Saarikallio et al., 2018; Stewart et al., 2019). This leads to emotional well-being and greater awareness (Dingle et al., 2016), which implies achieving a greater AMA. Therefore, there is the possibility to develop EI from musical strategies.

Regarding ASE, it is the group with high AMA that scores higher compared to the group with low AMA. Similarly, the results indicate that the probability of showing a high level of AMA increases as ASE increases.

According to previous studies, musical listening has a significant effect on positive affect, mental effort, and academic performance; however, it does not have a significant effect on perceived self-efficacy (Lim and Bang, 2018). In other words, a positive mood is able to be generated, which has an impact on the quality of a task and requires less mental effort and time spent on the task, without having a belief about higher ability (Lesiuk, 2005; Lim and Bang, 2018). However, other studies caution that, more concretely, ASE is linked to higher academic performance due to an individual having a belief about their ability to achieve an academic goal (Honicke and Broadbent, 2016; Yokoyama, 2019). There is no previous research examining the link between AMA and ASE, however, this study shows that feeling that one is capable of tackling an academic task has an impact on being more aware of one's own emotions generated by music, or at least thinking that one has a greater awareness of it. ASE is positively and significantly related to basic psychological needs, being based on competence, autonomy, and affinity needs, which contributes to optimal emotional development (Zhen et al., 2017), that is, perception, understanding, and clarity of feelings. Therefore, while there are studies that affirm the positive link established between EI and academic performance (Nirmala-Wijekoon et al., 2017) and even with quality of work-life, satisfaction, and work engagement (Karimi and Karimi, 2016), from this study, it can be stated that having the belief of greater academic ability allows one to be more aware of one's emotions, in this case musical ones. In other words, there is a greater chance of presenting more AMA.

Finally, in relation to the PATs, the group with a high level of AMA, vs. the group with low levels, scored higher in perfectionistic concerns, perfectionistic demands, and perfectionistic strivings. Furthermore, it is observed that the three PAT factors are also predictors of high AMA. In other words, the probability of high AMA increases as perfectionistic concerns, perfectionistic demands, and perfectionistic strivings increase.

In a recent study of PAT profiles of varying intensity, it was observed that the high PAT group scored higher in attention to feelings compared to the moderate and low PAT profiles. Moreover, understanding and clarity of feelings scored higher in the moderate PAT group followed by the high PAT profile (Aparicio-Flores et al., 2021). This may be due to the fact that the concern and demand to be perfect triggers a high degree of meticulousness with the purpose of achieving one's goals (Stoeber et al., 2009). This also causes a high level of alertness to one's own internal emotions. In other words, this leads to possessing greater attention to feelings, which generates greater understanding and emotion repair skills (Aparicio-Flores et al., 2021).

Despite there not being previous studies that determine a link between AMA and PATs, this study indicates that PATs, that is, perfectionistic concerns, perfectionistic demands, and perfectionistic strivings, all predict AMA. These findings may be due equally to the degree of meticulousness exhibited by individuals with PATs (Besharat and Kamali, 2016) that makes them alert to both their external and internal stimuli. On the one hand, perfectionistic concerns are worries about making mistakes. On the other hand, demands are based on the requirement for self-improvement to not only perform better but also to avoid possible negative situations. Likewise, efforts are defined by thoughts that are based on striving toward perfection (Stoeber et al., 2014; Esteve-Faubel et al., 2020). In other words, the three PAT factors, despite having their specific peculiarities, are governed by a continuous rumination toward reaching perfection or, on the contrary, toward not committing any imperfection. Besharat and Kamali (2016) stated that both PATs and ego control predicted meticulousness, which shows us that those with the presence of PATs need absolute control of their actions considering given the great psychological distress that they experience in the event of acting imperfectly or not performing in an impeccable way (Flett et al., 1998; Lyubomirsky et al., 2015). Hence, they are governed by meticulousness in all their internal and external actions, triggering greater EI (Aparicio-Flores et al., 2021), and consequently greater AMA.

### Limitations and Future Research

Several limitations should be noted. Firstly, despite having a large sample of participants, the results are not generalizable to the entire adolescent population. Likewise, due to the high sample of Spanish participants, they may find different cultural connotations. However, according to Argstatter (2015), it should be that at the level of emotional classification by musical stimuli, culture has no influence. Considering that our study is driven by AMA and not by attention to feelings after musical listening, it is likely that culture does not have a significant influence. However, in order to be able to affirm this hypothesis, it would be of great benefit to carry out a similar study with a population of similar ages that belong to different cultures in order to examine whether differences exist according to culture.

Moreover, this study shows differences in EI, ASE, and PATs according to high and low AMA, as well as showing that EI, ASE, and PATs are predictor variables of high AMA in late adolescence. However, there are no studies examining whether these results can be generalized to child and adult populations. Therefore, future studies should replicate the analyses performed with different age samples.

Furthermore, this work may be limited due to the use of self-report scales. This could be solved by employing an evaluation that uses multiple sources and methods.

### Practical Implications and Conclusions

Despite the aforementioned limitations, this is a novel study that contributes to the investigation of emotional, artistic, and academic aspects in the adolescent stage for several reasons. First, it is the first study to examine the differences in EI, ASE, and PATs according to high and low AMA, as well as examine whether these three variables predict high AMA. Secondly, the observed results provide specific insights into the emotional aspects of adolescents that may have a positive impact on their psychological well-being and academic performance. In other words, this direct link between these variables may be suggesting, as McGinnis (2017) stated, that teachers should orient their pedagogical perspective toward musical experiences that help the development of students' emotional self-regulation.

It is worth remembering that adolescence is framed as a stage in which very intense emotions are experienced, but also is one in which the skills to regulate them are not yet fully developed (Dingle et al., 2016). Music can have a positive influence in this, especially at this stage (Ciarrochi et al., 2011).

Music enhances both positive and negative emotions (Chong-Leung and Cheung, 2020), and being aware of the emotions it conveys can bring both internal and external benefits (Karimi, 2021). As such, it is important to include musical training in people's daily lives to consciously influence their psychological improvement and well-being (Lieberman et al., 2011; Chong-Leung and Cheung, 2020), which in turn will influence their academic performance (Lim and Bang, 2018) and quality of life (Boden et al., 2015).

For music to have special relevance in emotional education, further teacher training is urged both in the identification and regulation of emotions through musical listening, as well as in the management of specific music that allows working in the classroom depending on the situation and the feeling to be increased and/or analyzed (Lorenzo-de Reizábal, 2019).

## Data Availability Statement

The original contributions presented in the study are included in the article/supplementary material, further inquiries can be directed to the corresponding author/s.

## Ethics Statement

Ethical review and approval was not required for the study on human participants in accordance with the local legislation and institutional requirements. The patients/participants provided their written informed consent to participate in this study.

## Author Contributions

RE-F and MA-F: article conceptualization, investigation, writing original draft, and review and editing. VC-N: formal analysis, data curation, and supervision. JE-F: methodology, formal analysis, resources, data curation, supervision and project administration. All authors have read and agreed to the published version of the manuscript.

## Conflict of Interest

The authors declare that the research was conducted in the absence of any commercial or financial relationships that could be construed as a potential conflict of interest.

## Publisher's Note

All claims expressed in this article are solely those of the authors and do not necessarily represent those of their affiliated organizations, or those of the publisher, the editors and the reviewers. Any product that may be evaluated in this article, or claim that may be made by its manufacturer, is not guaranteed or endorsed by the publisher.
